# Laminin-332 γ2 Monomeric Chain Promotes Adhesion and Migration of Hepatocellular Carcinoma Cells

**DOI:** 10.3390/cancers15020373

**Published:** 2023-01-06

**Authors:** Rosanna Scialpi, Valentina Arrè, Gianluigi Giannelli, Francesco Dituri

**Affiliations:** National Institute of Gastroenterology IRCCS “Saverio de Bellis”, Research Hospital, Castellana Grotte, 70013 Bari, Italy

**Keywords:** HCC (hepatic cellular carcinoma), laminin 332 (Ln-332), Gamma 2 subunit, cell adhesion, cell migration, therapeutic target, cell scattering

## Abstract

**Simple Summary:**

Extracellular matrix (ECM) molecules are believed to being critically involved in the progression of hepatocellular carcinoma (HCC). Laminin332 (Ln332) is an ECM protein complex that has been found to be implicated in the acquisition of aggressive phenotype of HCC cancer cells. Here, we focus on the pro-invasive function of the γ2 chain of Ln332 in monomeric form, highlighting its ability to stimulate the motility of HCC cell, independent of and to the same extent as the entire Ln332. Our data provide proof of principle that blocking interactions between the γ2 chain and related receptors on the HCC cell surface may be strategic to counteract HCC aggravation.

**Abstract:**

Extracellular matrix (ECM) has a well-recognized impact on the progression of solid tumors, including hepatocellular carcinoma (HCC). Laminin 332 (Ln332) is a ECM molecule of epithelial basal lamina, composed of three polypeptide chains (α3, β3, and γ2), that is usually poorly expressed in the normal liver but is detected at high levels in HCC. This macromolecule was shown to promote the proliferation, epithelial-to-mesenchymal transition (EMT), and drug resistance of HCC cells. The monomeric γ2 chain is up-regulated and localized preferentially at the invasive edge of metastatic intrahepatic HCC nodules, suggesting its potential involvement in the acquisition of invasive properties of HCC cells. HCC cells were tested in in vitro adhesion, scattering, and transwell migration assays in response to fibronectin and the Ln332 and Ln332 γ2 chains, and the activation status of major signaling pathways involved was evaluated. Here, we show that the Ln332 γ2 chain promotes HCC the cell adhesion, migration, and scattering of HCC cells that express the Ln332 receptor α3β1 integrin, proving to be a causal factor of the EMT program achievement. Moreover, we found that efficient HCC cell adhesion and migration on γ2 require the activation of the small cytosolic GTPase Rac1 and ERKs signaling. These data suggest that the γ2 chain, independently from the full-length Ln332, can contribute to the pro-invasive potential of aggressive HCC cell subpopulations.

## 1. Introduction

Hepatocellular carcinoma (HCC) is the sixth most common malignancy and the third cause of cancer-related death worldwide. The prolonged intake of alcohol or aflatoxin; a sedentary lifestyle; a fatty diet leading to obesity, and chronic infection with hepatitis B and C virus (HBV, HCV) are recognized as etiological factors for the onset of HCC [1,2]. The composition and abundancy of macromolecules that build up the tumor microenvironment critically influence the biology of solid cancers, including HCC, as they affect some properties of cancer cells, such as invasion and metastatic dissemination [3].

Hepatic stellate cells (HSCs) are the primary source of ECM deposition, in particular following their terminal differentiation into myofibroblasts in response to a variety of stimuli [4]. One of the main abundant components of the ECM product of HSCs is laminin-332 (Ln332) [5]. Ln332 is a heterotrimeric glycosylated protein, a member of the Ln family, formed by three chains assembled in a coiled structure with disulfide bounds and α3, β3, and γ2 chains, respectively [6]. In a healthy liver, Ln332 is virtually absent, but it is abundant in the HCC tissue, where it has been reported to support proliferation, migration, and invasion of tumor cells [7]. Ln332 may support the progression of solid tumors, such as HCC, through the interaction of its α3 chain with α3β1 and α6β4 integrins, which, in turn, activate various downstream signaling mediators, such as focal adhesion kinase, phosphoinositide 3-kinase, and integrin-linked kinase [8,9,10]. In HCC, Ln332 was reported to act synergistically with transforming growth factor beta (TGFβ) to activate the EMT program [11].

The γ2 chain of Ln332 likely plays a crucial role in cancer invasion and metastasis as it is deposited at high concentration at the tumor invading edge of solid tumors, and it mediates the migration and invasion of transformed cells of cancers, including gastric and pancreatic cancer [12,13].

Human Ln332 γ2 is synthesized and secreted as a precursor 150 kDa. After secretion, it undergoes specific extracellular proteolytic processing and is converted to the mature form 105 kDa [14]. In rats, the γ2 chain of Ln332 is processed from the 150 kDa form to the 80-kDa mature and active form by matrix metalloproteinase [MMP]-2 or membrane type 1-MMP, and this maturation enhances the cell motility activity of the Ln332 [15,16].

In this study, we test the proficiency of human recombinant monomeric Ln332 γ2 in promoting HCC cell adhesion and migration, in comparison with human recombinant full-length Ln332, and gain insights into the activation status of the signaling mediators and effectors involved.

## 2. Materials and Methods

### 2.1. Cell Culture and Reagents

HLE and HLF cell lines were purchased from JCRB Cell Bank (Japan) and cultured in DMEM (Dulbecco’s Modified Eagle Medium) supplemented with antibiotic–antimycotic, sodium pyruvate, 10% fetal bovine serum (FBS), and HEPES (Thermo Fisher Scientific, Waltham, MA, USA). Cells were tested for the absence of mycoplasma contamination using the MycoFluor™ Mycoplasma Detection Kit (Thermo Fisher Scientific). Full-size recombinant human laminin-332 was purchased from Kerafast (Boston, MA). Fibronectin (from human plasma) was purchased from Millipore (Temecula, CA, USA). Recombinant Ln332 γ2 chain was purified as previously described [17]. Extracellular signal-regulated kinases (ERKs) inhibitor (PD98059) and Rac1 inhibitor (CAS 1177865-17-6) were purchased from Merck (Merck, Germany). Protein kinase B (PKB, or Akt) inhibitor (LY294002) was purchased from Selleckchem (Houston, TX, USA). Anti-phospho/total-Akt and anti-phospho/total-ERKs antibodies were purchased from cell signaling technology (Danvers, MA, USA).

### 2.2. Adhesion Assay

Adhesion assay was performed as previously described [18]. Briefly, wells of a 96 well plate were coated with 2% bovine serum albumin (BSA, Sigma Aldrich, St. Louis, MI, USA), Fn (10 µg/mL), Ln332 (10 µg/mL), or γ2 (3.125 µg/mL), diluted in phosphate buffered saline (PBS) for 2 h at 37 °C. All wells were then washed three times with PBS, covered with BSA solution to coat any residual uncoated areas, and incubated for 1 h at 37 °C. The wells were finally washed three times with PBS and allowed to dry out for a few minutes. In total, 35,000 cells were diluted in 100 µL of serum-free DMEM medium +0.5% BSA, seeded onto BSA-, Fn-Ln332-, or Ln332-γ2-coated wells, and then incubated at 37 °C, 5% CO_2_ for 30 minutes. An equivalent volume of 4% paraformaldehyde (PFA, pH 7.2 in PBS) was added, and the plates were immediately flicked for a few seconds to allow the mixing and incubated for 30 min at room temperature. The medium + PFA was then removed, and adherent cells were stained with Cristal Violet for 10 min. After abundant washing with distilled water, the stained cells were allowed to dry out and solubilized with 100 µL of a 1% sodium dodecyl sulfate solution. Absorbance was read at 595 nm and proportionally related to the number of adhered cells.

### 2.3. Transwell Migration Assay

The assay was performed as previously described [19]. Briefly, 15,000 cells were suspended in 200 µL of serum-free DMEM medium containing 0.5% BSA loaded onto the top chamber of polycarbonate transwell inserts (suitable for a 24-well plate, a 6.5 mm internal diameter, and an 8 µm pore size, Corning, NY, USA), the membrane of which had been previously coated for 2 h at room temperature with fibronectin (Fn at concentration 10 µg/mL), Ln332, and Ln332 γ2 on the lower surface. Cells were allowed to migrate for 16 h in serum-free DMEM medium + 0.5% BSA at 37 °C and 5% CO_2_, then fixed in 4% PFA (pH 7.2 in PBS) and stained with crystal violet for 10 min. Five fields per membrane were captured at 100× (490 × 690 µm area, bright field) or 200× (245 × 345 µm area, bright field) magnification, and the number of cells/field was counted.

### 2.4. Scattering Assay

HLE and HLF cells were plated on 24-well plate at low density, incubated (37 °C, 5% CO_2_), and allowed to form tiny islets. A few days later, cells were serum-starved and incubated in the presence/absence of Ln332 (10 µg/mL), Ln332 γ2-chain (3.125 µg/mL), or hepatic growth factor (HGF, 100 ng/mL). Microscopic images were acquired at 0 h, 12 h, and 24 h time points (100× magnification, phase contrast, and 490 × 690 µm area).

### 2.5. Microscopy Analyses

All the microscopic images were acquired using Nikon Eclipse Ti2 microscope equipped with color camera Nikon DS-Fi3. The objectives used were 10× achromatic objective, numerical aperture 0.25; 20× Plan Fluor dichroic N2, numerical aperture 0.5. Analyses were performed using the software NIS-Elements (version 5.11.01).

### 2.6. Western Blot

Cell proteins were extracted using 1× lysis/binding/wash buffer (active Rac1 pull-down and detection kit, Thermo Fisher Scientific) with Halt protease and phosphatase inhibitor cocktail EDTA-free (Thermo Fisher Scientific). Briefly, the lysates were incubated on ice for 10 minutes and vortexed every 5 min. Then, the samples were clarified through centrifugation at 16,000× *g* (at 4 °C) for 15 min to precipitate insoluble debris. The supernatants (containing the extracted proteins) were assayed for protein concentration using Bradford Reagent (Bio-Rad). The proteins were then mixed with Laemmli buffer 4× and 10% β-mercapto ethanol, and denatured at 95 °C for 5 min. In total, 25 µg of total proteins was loaded onto 4–20% polyacrylamide gradient gels and run in sodium dodecyl sulfate polyacrylamide gel electrophoresis. After separation, the proteins were transferred onto the PVDF membrane (Trans-Blot Turbo Mini 0.2 µm PVDF Transfer Packs, Bio-Rad) using the trans-blot turbo transfer system (Bio-Rad), stained with primary and horseradish peroxidase-conjugated secondary antibodies, and revealed using the clarity max western ECL substrate (Bio-Rad).

### 2.7. Active Rac1 Pull-Down Assays

Active Rac1 pull-down assays were conducted in accordance with the manufacturer’s protocols (active Rac1 pull-down and detection kit, Thermo Fisher Scientific). Rac1-GTP and total Rac1 were detected using the antibody provided by the same kit.

### 2.8. Statistical Analysis

The two-tailed t-Student test was used to measure the statistical significance for differences between the groups. *p*-values of less than 0.05 were considered statistically significant. Statistical analyses were performed using Prism (GraphPad, Dotmatics).

## 3. Results

### 3.1. Soluble γ2 Chain of Ln332 Promotes HCC Cell Adhesion, Scattering, and Migration

**Figure 1:**
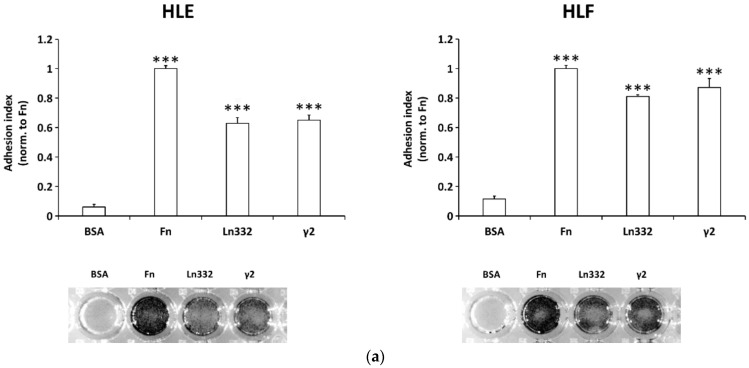

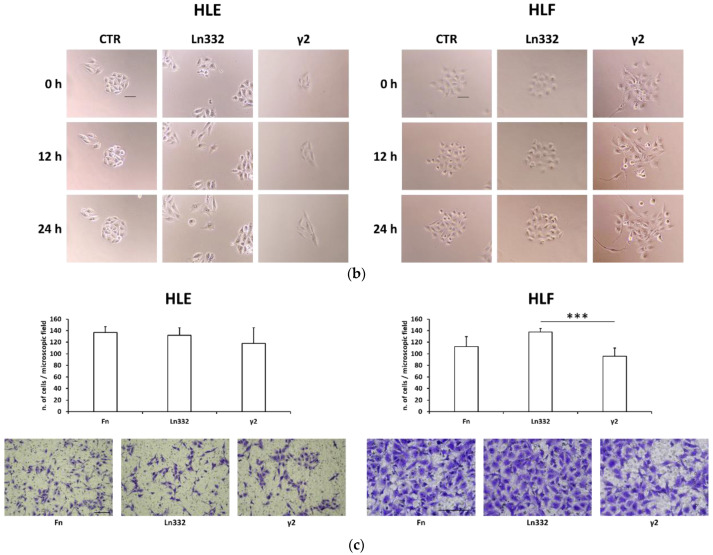
Laminin-332 γ2 chain promotes adhesion, scattering, and migration of HCC cells. (**a**) Cell adhesion assay. HLE or HLF cells were seeded on uncoated (BSA), fibronectin (Fn), laminin-332 (Ln332), or γ2 chain (γ2)-coated well surfaces and allowed to attach and spread for 30 minutes. Data are the means ± SD of triplicates; (**b**) scattering assay (File S1). Colonies made by 8–10 cells of HLE and HLF cells were left untreated or treated with soluble Ln332 (10 µg/mL) and γ2 (3.125 µg/mL) in serum free medium for 24 h; scale bar = 50 µm; a replicate experiment, including stimulation with hepatocyte growth factor (HGF, at the concentration of 100 ng/mL) as a positive control condition, is shown in Figure S1; (**c**) transwell migration assay. HLE and HLF cells were seeded on the top of the transwell membrane, previously coated with Fn, Ln332, or γ2 on the lower side and allowed to migrate for 16 h. Data are the means ± SD of five randomly chosen microscopic fields. T test (unpaired, two-tailed), *** *p* < 0.001 (vs. BSA in panel (**a**)); and scale bar = 100 µm.

While it is well established that Ln332, or its processed forms as a result of cleavage by metalloproteinases, promotes the invasion and migration of HCC cells, the role of Ln332 γ2 fragment resulting from these enzymatic activities has not been directly assessed. Cell adhesion to ECM proteins supposed to drive motility is a prerequisite for subsequent migration. The propensity of HLE and HLF HCC cell lines to perform adhesion, migration, and scattering on Ln332 and Ln332 γ2 was investigated. Cells were challenged for adhesion and migration onto surfaces coated with Ln332 or Ln332 γ2. BSA and fibronectin were used as a negative and a positive control, respectively, in all the assays performed. Ln332 and Ln332 γ2 were used in stoichiometric proportion to allow a direct comparison of their biological activity. We found that the adhesion and spreading of HLE and HLF cells on Ln332 and Ln332 γ2 was promoted 30 min after the seeding, suggesting the involvement of a integrin-mediated cell-matrix interaction (Figure 1a). Cell scattering, which intervenes as a result of the down-regulation of cell–cell direct interaction mediated by cadherin engagement, suggests an acquired aptitude of cells to migrate and invade. To assess whether Ln332 and Ln332 γ2 are implicated in the occurrence of this phenomenon, we allowed HCC cells to form small islets and then exposed them to both macromolecules in the soluble form for 24 h. We found that Ln332 γ2, as well as Ln332, enhances cell scattering after 24 h (Figure 1b). Surprisingly, in a transwell assay, Ln332 and γ2 were both as efficient as fibronectin in inducing migration, confirming the powerful pro-migratory capacity of Ln332 and, more interestingly, of its γ2 fragment (Figure 1c). Nevertheless, Ln332-induced migration tended to be slightly increased compared to that induced by γ2, probably suggesting additive functional roles of domains present in other chains of Ln332 but not in the γ2 chain (such as the integrin-binding G domain of the α3 chain).

### 3.2. Ln332 γ2 Chain Activates Pro-Migratory Signaling Pathway in HCC Cells

**Figure 2:**
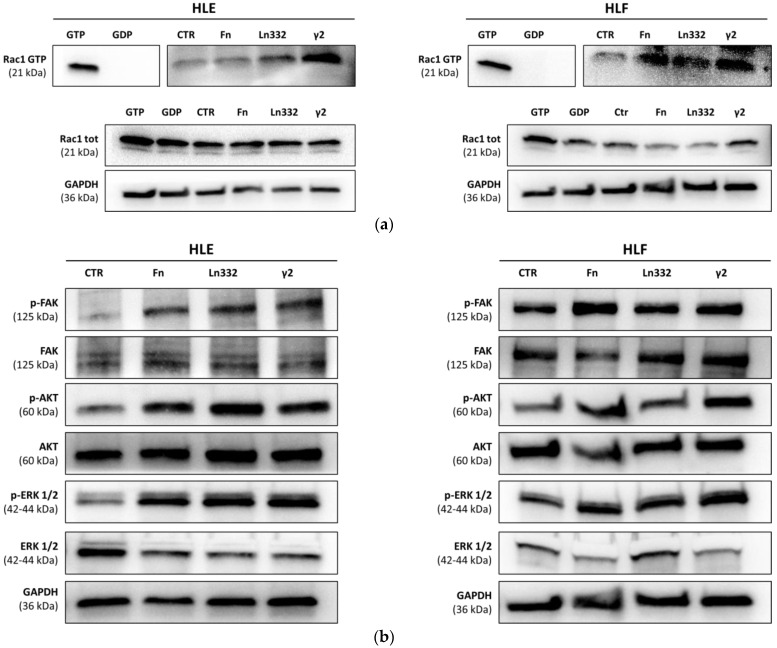
Adhesion of HCC cells onto γ2 chain of Ln332 promotes activation of pro-migratory signaling mediators. (**a**) Western blot analysis for active Rac1 (Rac1-GTP) protein levels in HLE and HLF upon seeding and 30 min spreading on uncoated, fibronectin (Fn), Ln332, or γ2-coated surfaces. (**b**) Western blot analysis for phospho-FAK, phospho-Akt, and phospho-ERKs in HLE and HLF cells upon seeding and 30 min spreading on uncoated, Fn, Ln332, or γ2-coated surfaces. These experiments were performed in replicate (see Figure S2 for row blots).

After establishing that Ln332 γ2 chain, as well as full-length Ln332, promotes the adhesion and migration of HCC cells, we investigated the molecular pathway involved. Focal signaling hubs regulate the dynamics of assembly and disassembly of cytoskeletal structures required for cancer cells to lose contact with primary tumor nodules and metastasize. The activation of small intracellular GTPases of the Rho family (including Rac1, Cdc42, and Rho itself) represents a well-established biochemical readout for the cell motility phenotype [20]. GTPases Rho and Rac regulate the assembly of actin filament and the formation of integrin adhesion complexes to produce stress fibers and lamellipodia [21,22]. Rac1 plays a determinant role in regulating the actin cytoskeleton by activating p21-activated kinases, enhancing cell proliferation through the MAPK (mitogen-activated protein kinase) system. These functions of Rac1 become crucial for angiogenesis and tumor promotion, invasion, and metastasis [23,24,25]. Based on these assumptions, we tested whether the binding of Ln332 γ2 to the surface of HLE and HLF cells is able to induce the activation of Rac1. After 30 min of adhesion on surfaces coated with fibronectin, Ln332, or Ln332 γ2, the GTP-bound active fraction of Rac1 increased (Figure 2a). The phosphorylation of focal adhesion kinase, Akt, and ERK1/2 also increased in the same condition of adhesion (Figure 2b). This evidence further supports the pro-migratory activity of γ2 chain as a monomer, independently of the fully-assembled Ln332 complex.

### 3.3. Adhesion and Spreading of HCC Cells on γ2 Chain of Laminin-332 depends on ERKs and Rac1 Activation

**Figure 3:**
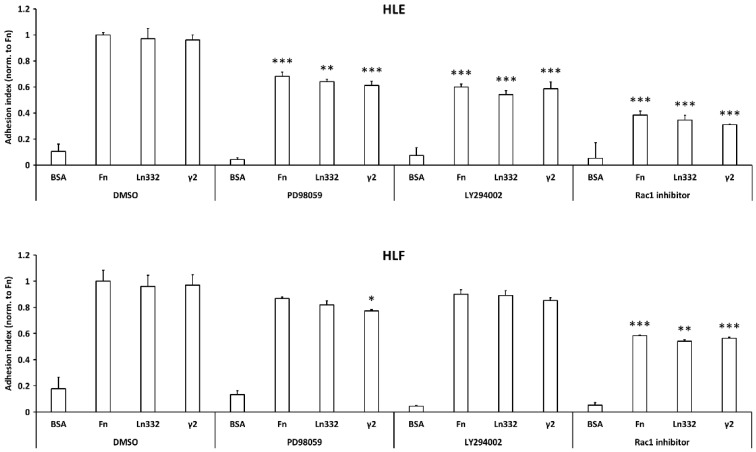
Adhesion of HCC cells to γ2 chain of Ln332 depends in part on the activation of Rac1. HLE and HLF were pre-incubated with vehicle (DMSO, dilution 1:286 vol/vol), ERK1/2 inhibitor (PD98059, 50 μM), AKT inhibitor (LY294002, 25 μM), or active Rac1 inhibitor (CAS 117786517-6, 50 μM), for 2 h at 37 °C; then, the cells were seeded on uncoated, or fibronectin (Fn), or laminin-332 (Ln332), or γ2 -coated well surfaces and allowed to attach and spread for 30 minutes. Data are the means ± SD of triplicates, T test (unpaired, two-tailed): * *p* < 0.05, ** *p* < 0.01, and *** *p* < 0.001, vs. corresponding ECM protein in control (DMSO).

Having found that ERK1/2, AKT, and Rac1 become more activated upon the binding of the Ln332 γ2 chain on the HCC cell surface, we investigated whether the activation of these signaling mediators is required for HCC cells to perform adhesion and spreading on γ2. Cells were pre-treated with vehicle (DMSO), or the inhibitors of MEK (PD98059), Akt (LY294002), or Rac1 (CAS 117786517-6), for two hours, and challenged for 30 min adhesion onto surfaces coated with BSA (negative control), fibronectin (positive control), Ln332, and Ln332 γ2. Ln332 and Ln332 γ2 were used in stoichiometric proportion to allow for a direct comparison of their biological activity. We found that the adhesion of HLE and HLF cells on all three ECM proteins was reduced in the presence of Rac1 inhibition, whereas MEK blockage was effective on the adhesion of both cell lines limited to Ln332 γ2. These data suggest the involvement of ERK1/2 and Rac1 in cell attachment and spreading upon interaction with Ln332 γ2 (Figure 3).

### 3.4. Pro-Migratory Activity of Ln332 γ2 depends upon MEK-ERK1/2 Pathway Activation

**Figure 4:**
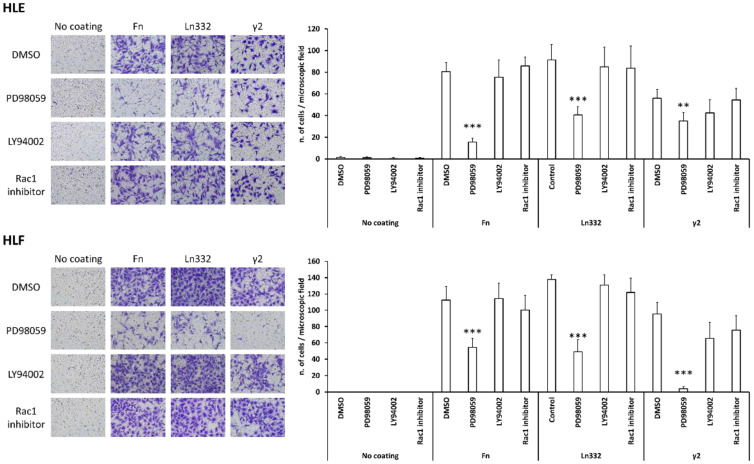
Migration of HCC cells to fibronectin, Ln332, and Ln332 γ2 chains depends on the activation of ERK1/2. HLE and HLF cells were seeded on the top of the transwell membrane, previously coated with Fn, or Ln332, or γ2, on the lower side and allowed to migrate for 16 h in culture medium serum free with vehicle (DMSO, diluted 1:286 vol/vol), MEK inhibitor (PD98059, 50 μM), AKT inhibitor (LY294002, 25 μM), or active Rac1 inhibitor (CAS 117786517-6, 50 μM). Data are the means ± SD of five randomly chosen microscopic fields. T test (unpaired, two-tailed): ** *p* < 0.01, and *** *p* < 0.001 vs. control (DMSO) of related ECM protein coating condition. Scale bar = 100 µm.

To assess whether the activity of the signaling mediators (ERK1/2, AKT, and Rac1) taken into consideration for HCC cell adhesion on Ln332 γ2 may also be required for migration on this chain, we set up a 16 h’ migration transwell assay of HLE and HLF cells on fibronectin, Ln332, and Ln332 γ2 in the absence/presence of inhibitors of MEK, AKT, and Rac1 at the same concentrations as used in adhesion assay. As a result, the blockade of MEK-ERK1/2 activation led to a marked reduction in migratory activity of HLE and HLF on all three ECM proteins, while the inhibition of Akt and Rac1 was ineffective (Figure 4). According to these data, HCC cells mainly rely on Rac1 activity for adhesion, while ERKs function serve to effective motility on γ2 chain.

## 4. Discussion

The present study demonstrates that the γ2 chain of Ln332 promotes HCC cell adhesion, migration, and scattering in both soluble and insoluble (coated) forms to a similar extent as fully assembled Ln332 and fibronectin. While the adhesiveness of HCC cells on γ2 is dependent, at least in part, upon the activation status of ERKs and Rac1, only ERKs appear to mediate HCC cell migration through γ2. This discrepancy may reflect the requirement of differential biochemical intracellular signals for HCC cells to perform adhesion and subsequent motility, as well as the distant interdependence of the two processes. The ability of γ2 to promote adhesion, scattering, and migration can be explained by the presence of multiple functional domains located on this chain and interactions taking place among γ2 and various cell surface receptors, including growth factor receptors, integrins and proteoglycans. The processing of the γ2 chain within the entire Ln332 complex by MMP-2 cleavage exposes cryptic pro-migratory sites that induce the migration of the breast epithelial cell [15]. Furthermore, the cryptic EGF-like domain III of γ2 binds and activates the EGF receptor to stimulate cell migration [26,27]. In addition, the short arm of the γ2 chain, γ2sa (resulting from proteolytic cleavage), was found to bind to syndecan-1 via its domain V and to enhance cell adhesion while suppressing motility [14].

Unlike the other two chains composing Ln332, γ2 has the particularity of being the only chain of the Ln332 complex that can be secreted as a monomeric form [28]. In human carcinomas, the Ln332 γ2 chain is highly expressed by cells that exhibit an invading phenotype [29,30]. Human gastric carcinoma cells secrete a high level of Ln γ2 chain monomer, in addition to the Ln332 complex, into culture medium. Tumor cells at the invading fronts showed strong cytoplasmic staining for the Ln γ2 chain without any detectable signal for the α3 and β3 chains of Ln322 in both well and poorly differentiated carcinomas. In differentiated carcinomas, the basement membranes surrounding neoplastic glandular structures show intense and continuous immunoreactivity for the Ln332 γ2. Contrariwise, the organization of this chain appears irregular and scattered in poorly differentiated carcinomas [12]. In HCC, γ2 is detected in tight association with invading edge of intrahepatic metastatic nodules [7]. Interaction between γ2 and membrane-type 1 metalloproteinase (MT1-MMP) was found to positively correlate with both metastasis occurrence and the degree of invasion in esophageal squamous cell carcinomas [31]. In addition, circulating γ2 fragments in serum are elevated in pancreatic ductal adenocarcinoma patients with liver metastases, suggesting that circulating γ2 fragment may be useful as a prognostic indicator [32].

Many intracellular signaling mediators, including mitogen-activated protein kinases (MAPKs), phosphatidylinositol-3-kinase (PI3K)/Akt, and small cytoplasmic GTPases, are involved in cytoskeleton dynamics that may affect migration; invasion; and, ultimately, the metastatic evolution of solid cancers, including HCC [33,34,35,36,37,38,39]. The Ln332 complex was found to activate the motility of HCC cells via MEK/ERK signaling [5]. Consistently, we found that the same pathway is required for the migration of these cells on monomeric γ2.

## 5. Conclusions

In conclusion, we provide evidence that Ln332 γ2 can promote HCC cell adhesion and migration, both in solution or in coating, regardless of whether it is incorporated into the Ln332 complex or not. This proof of concept may be of interest from the perspective of designing new potential agents to specifically target this peptide for the treatment of HCC.

## Data Availability

The data presented in this study are available on request from the corresponding author.

## Supplementary Materials

The following are available online at www.mdpi.com/article/10.3390/cancers15020373/s1, Figure S1: Replicate of scattering assays (related to Figure 1b) including positive control (scale bar = 50 µm), Figure S2: Raw blots and densitometry quantification related to Figure 2, File S1: Movies of scattering assays related to Figure 1b.

## References

[1] Ferlay J., Soerjomataram I., Dikshit R., Eser S., Mathers C., Rebelo M., Parkin D.M., Forman D., Bray F. (2015). Cancer incidence and mortality worldwide: Sources, methods and major patterns in GLOBOCAN 2012. Int. J. Cancer.

[2] Forner A., Llovet J.M., Bruix J. (2012). Hepatocellular carcinoma. Lancet.

[3] Rani B. (2014). Role of the tissue microenvironment as a therapeutic target in hepatocellular carcinoma. World J. Gastroenterol..

[4] Pinzani M., Macias-Barragan J. (2010). Update on the pathophysiology of liver fibrosis. Expert Rev. Gastroenterol. Hepatol..

[5] Santamato A., Fransvea E., Dituri F., Caligiuri A., Quaranta M., Niimi T., Pinzani M., Antonaci S., Giannelli G. (2011). Hepatic stellate cells stimulate HCC cell migration via laminin-5 production. Clin. Sci..

[6] Burgeson R.E., Chiquet M., Deutzmann R., Ekblom P., Engel J., Kleinman H., Martin G.R., Meneguzzi G., Paulsson M., Sanes J. (1994). A new nomenclature for the laminins. Matrix Biol..

[7] Giannelli G., Fransvea E., Bergamini C., Marinosci F., Antonaci S. (2003). Laminin-5 chains are expressed differentially in metastatic and nonmetastatic hepatocellular carcinoma. Clin. Cancer Res..

[8] Bergamini C., Sgarra C., Trerotoli P., Lupo L., Azzariti A., Antonaci S., Giannelli G. (2007). Laminin-5 stimulates hepatocellular carcinoma growth through a different function of α6β4 and α3β1 integrins. Hepatology.

[9] Korkut A., Zaidi S., Kanchi R.S., Rao S., Gough N.R., Schultz A., Li X., Lorenzi P.L., Berger A.C., Robertson G. (2018). A Pan-Cancer Analysis Reveals High-Frequency Genetic Alterations in Mediators of Signaling by the TGF-β Superfamily. Cell Syst..

[10] Eke I., Cordes N. (2015). Focal adhesion signaling and therapy resistance in cancer. Semin. Cancer Biol..

[11] Giannelli G., Bergamini C., Fransvea E., Sgarra C., Antonaci S. (2005). Laminin-5 with Transforming Growth Factor-β1 Induces Epithelial to Mesenchymal Transition in Hepatocellular Carcinoma. Gastroenterology.

[12] Koshikawa N., Moriyama K., Takamura H., Mizushima H., Nagashima Y., Yanoma S., Miyazaki K. (1999). Overexpression of laminin gamma2 chain monomer in invading gastric carcinoma cells. Cancer Res..

[13] Chen J., Zhang H., Luo J., Wu X., Li X., Zhao X., Zhou D., Yu S. (2018). Overexpression of α3, β3 and γ2 chains of laminin-332 is associated with poor prognosis in pancreatic ductal adenocarcinoma. Oncol. Lett..

[14] Ogawa T., Tsubota Y., Hashimoto J., Kariya Y., Miyazaki K. (2007). The short arm of laminin gamma2 chain of laminin-5 (laminin-332) binds syndecan-1 and regulates cellular adhesion and migration by suppressing phosphorylation of integrin beta4 chain. Mol. Biol. Cell.

[15] Giannelli G., Falk-Marzillier J., Schiraldi O., Stetler-Stevenson W.G., Quaranta V. (1997). Induction of cell migration by matrix metalloprotease-2 cleavage of laminin-5. Science.

[16] Koshikawa N., Giannelli G., Cirulli V., Miyazaki K., Quaranta V. (2000). Role of Cell Surface Metalloprotease Mt1-Mmp in Epithelial Cell Migration over Laminin-5. J. Cell Biol..

[17] Koshikawa N., Schenk S., Moeckel G., Sharabi A., Miyazaki K., Gardner H., Zent R., Quaranta V. (2004). Proteolytic processing of laminin-5 by MT1-MMP in tissues and its effects on epithelial cell morphology. FASEB J..

[18] Dituri F., Scialpi R., Schmidt T.A., Frusciante M., Mancarella S., Lupo L.G., Villa E., Giannelli G. (2020). Proteoglycan-4 is correlated with longer survival in HCC patients and enhances sorafenib and regorafenib effectiveness via CD44 in vitro. Cell Death Dis..

[19] Fransvea E., Angelotti U., Antonaci S., Giannelli G. (2008). Blocking transforming growth factor-beta up-regulates E-cadherin and reduces migration and invasion of hepatocellular carcinoma cells. Hepatology.

[20] Vilchez Mercedes S.A., Bocci F., Levine H., Onuchic J.N., Jolly M.K., Wong P.K. (2021). Decoding leader cells in collective cancer invasion. Nat. Rev. Cancer.

[21] Mackay D.J.G., Esch F., Furthmayr H., Hall A. (1997). Rho- and Rac-dependent Assembly of Focal Adhesion Complexes and Actin Filaments in Permeabilized Fibroblasts: An Essential Role for Ezrin/Radixin/Moesin Proteins. J. Cell Biol..

[22] Nethe M., Hordijk P.L. (2010). The role of ubiquitylation and degradation in RhoGTPase signalling. J. Cell Sci..

[23] Yadav S., Barton M.J., Nguyen N.-T. (2019). Biophysical properties of cells for cancer diagnosis. J. Biomech..

[24] Kazanietz M.G., Caloca M.J. (2017). The Rac GTPase in Cancer: From Old Concepts to New Paradigms. Cancer Res..

[25] Bustelo X.R. (2018). RHO GTPases in cancer: Known facts, open questions, and therapeutic challenges. Biochem. Soc. Trans..

[26] Schenk S., Hintermann E., Bilban M., Koshikawa N., Hojilla C., Khokha R., Quaranta V. (2003). Binding to EGF receptor of a laminin-5 EGF-like fragment liberated during MMP-dependent mammary gland involution. J. Cell Biol..

[27] Schenk S., Quaranta V. (2003). Tales from the crypt[ic] sites of the extracellular matrix. Trends Cell Biol..

[28] Gagnoux-Palacios L., Allegra M., Spirito F., Pommeret O., Romero C., Ortonne J., Meneguzzi G. (2001). The Short Arm of the Laminin γ2 Chain Plays a Pivotal Role in the Incorporation of Laminin 5 into the Extracellular Matrix and in Cell Adhesion. J. Cell Biol..

[29] Pyke C., Rømer J., Kallunki P., Lund L.R., Ralfkiaer E., Danø K., Tryggvason K. (1994). The gamma 2 chain of kalinin/laminin 5 is preferentially expressed in invading malignant cells in human cancers. Am. J. Pathol..

[30] Pyke C., Salo S., Ralfkiaer E., Rømer J., Danø K., Tryggvason K. (1995). Laminin-5 is a marker of invading cancer cells in some human carcinomas and is coexpressed with the receptor for urokinase plasminogen activator in budding cancer cells in colon adenocarcinomas. Cancer Res..

[31] Shen X.-M., Wu Y.-P., Feng Y.-B., Luo M.-L., Du X.-L., Zhang Y., Cai Y., Xu X., Han Y.-L., Zhang X. (2007). Interaction of MT1-MMP and laminin-5gamma2 chain correlates with metastasis and invasiveness in human esophageal squamous cell carcinoma. Clin. Exp. Metastasis.

[32] Katayama M., Funakoshi A., Sumii T., Sanzen N., Sekiguchi K. (2005). Laminin gamma2-chain fragment circulating level increases in patients with metastatic pancreatic ductal cell adenocarcinomas. Cancer Lett..

[33] Bourne H.R., Sanders D.A., McCormick F. (1991). The GTPase superfamily: Conserved structure and molecular mechanism. Nature.

[34] Hall A. (1994). Small GTP-binding proteins and the regulation of the actin cytoskeleton. Annu. Rev. Cell Biol..

[35] Reddy K.B., Nabha S.M., Atanaskova N. (2003). Role of MAP kinase in tumor progression and invasion. Cancer Metastasis Rev..

[36] Shih Y.-W., Chen P.-S., Wu C.-H., Jeng Y.-F., Wang C.-J. (2007). Alpha-chaconine-reduced metastasis involves a PI3K/Akt signaling pathway with downregulation of NF-kappaB in human lung adenocarcinoma A549 cells. J. Agric. Food Chem..

[37] Gupta A.K., Soto D.E., Feldman M.D., Goldsmith J.D., Mick R., Hahn S.M., Machtay M., Muschel R.J., McKenna W.G. (2004). Signaling pathways in NSCLC as a predictor of outcome and response to therapy. Lung.

[38] Osaki M., Oshimura M., Ito H. (2004). PI3K-Akt pathway: Its functions and alterations in human cancer. Apoptosis.

[39] Vivanco I., Sawyers C.L. (2002). The phosphatidylinositol 3-Kinase–AKT pathway in human cancer. Nat. Rev. Cancer.

