# Supplementary figures and images for: Laminin-332 γ2 Monomeric Chain Promotes Adhesion and Migration of Hepatocellular Carcinoma Cells
Source: Cancers (Basel). 2023 Jan 6;15(2):373. doi: 10.3390/cancers15020373 (PMC9857196; doi:10.3390/cancers15020373)

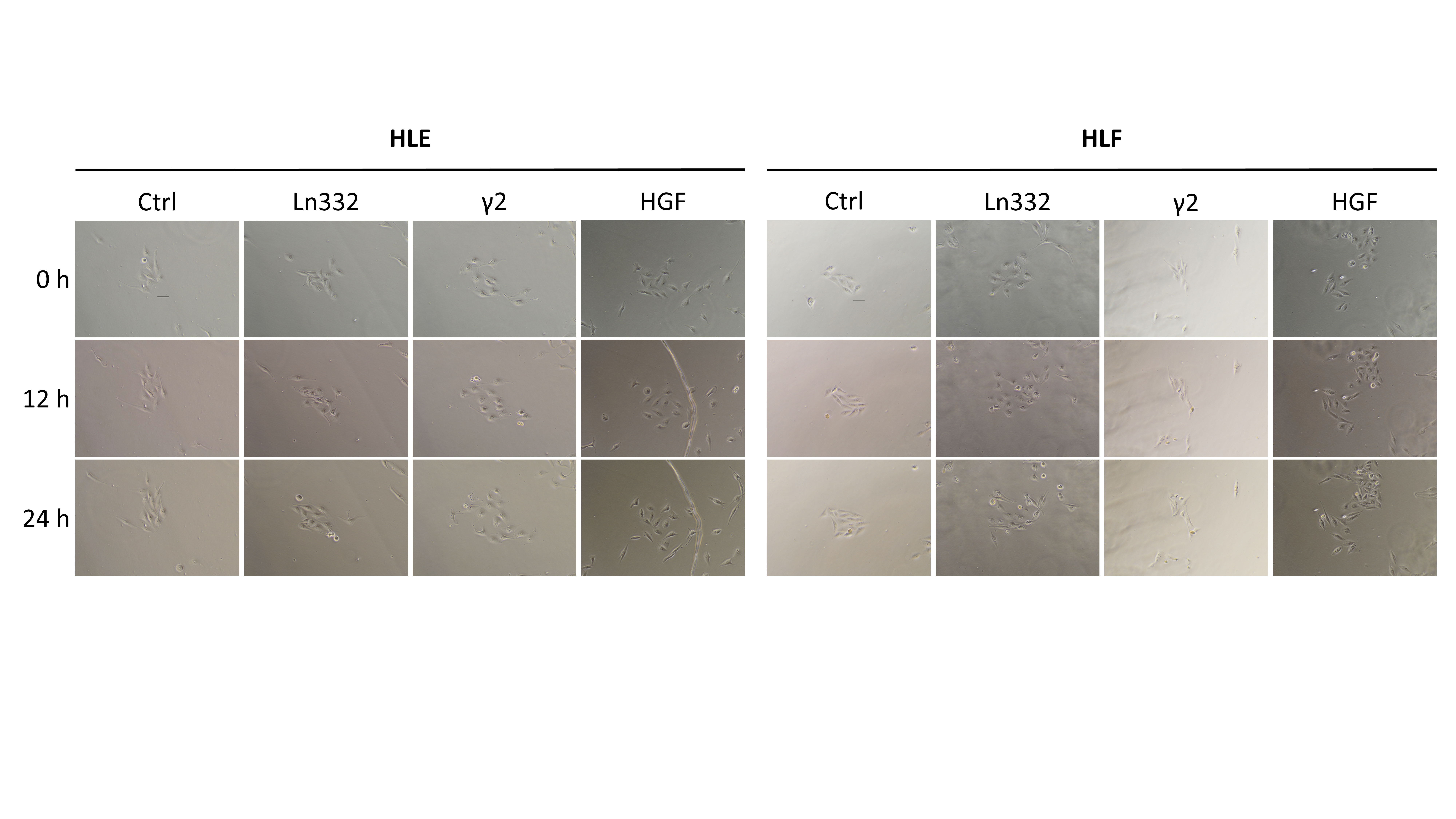

Supplement: Supplementary file 1 [file cancers-15-00373-s001.zip › Figure S1.tif]
